# Supplementary material for: Identification and classification of factors affecting the non-use of safety harness at height among construction workers in Tehran
Source: PLoS One. 2023 Nov 30;18(11):e0287906. doi: 10.1371/journal.pone.0287906 (PMC10688616; doi:10.1371/journal.pone.0287906)
Supplement: Attachment — Submitted filename: Response to Reviewers.doc [file pone.0287906.s002.doc]

**Dear Mohammad Hossein Ebrahimi**

**Editorial Section Manager**

**PLOS ONE**

Thank you for your attention in reviewing our paper and providing valuable comments. We have carefully reviewed your comments and have revised the manuscript accordingly. Here are some of the changes we have made in response to the reviewers' and editorial comments and questions.

**Editor comments:**

**Comment:**

1. Please ensure that your manuscript meets PLOS ONE's style requirements, including those for file naming.

**Answer:**

Our paper followed all PLOS ONE style guidelines, including those pertaining to file naming. We confirm that our manuscript adheres to the style requirements of the PLOS ONE, including file-naming conventions. -----------------------------------------------------------

**Comment:**

2. In the Methods section, please provide additional details regarding participant consent. In the ethics statement in the Methods and online submission information, please ensure that you have specified what type you obtained (for instance, written or verbal, and if verbal, how it was documented and witnessed). If your study included minors, state whether you obtained consent from parents or guardians. If the need for consent was waived by the ethics committee, please include this information. These items were added and highlighted in the method.

**Answer:**

The study protocol was reviewed and approved by the Research Ethics Committees of the School of Public Health and Neuroscience Research Center, Shahid Beheshti University of Medical Sciences, Tehran. Iran, with Approval ID: R. SBMU.PHNS.REC.1401.083. All the workers who participated in this study completed a written consent form. The age requirement for inclusion in the study was 20 to 50 years old. These items were added and highlighted in the method.

------------------------------------------------------------

**Comment:**

3. Thank you for stating the following financial disclosure:

“NO - Include this sentence at the end of your statement: The funders had no role in study design, data collection and analysis, decision to publish, or preparation of the manuscript”

At this time, please address the following queries:

a)        Please clarify the sources of funding (financial or material support) for your study. List the grants or organizations that supported your study, including funding received from your institution.

b)        State what role the funders took in the study. If the funders had no role in your study, please state: “The funders had no role in study design, data collection and analysis, decision to publish, or preparation of the manuscript.”

c)        If any authors received a salary from any of your funders, please state which authors and which funders.

d)        If you did not receive any funding for this study, please state: “The authors received no specific funding for this work.”

Please include your amended statements within your cover letter; we will change the online submission form on your behalf.

**Answer:**

Mousa Jabbari was supported by Shahid Beheshti University of Medical Sciences [31938]. The funders had no role in the study design, data collection and analysis, decision to publish, or manuscript preparation

**Comment:**

**4.** In your Data Availability statement, you have not specified where the minimal data set underlying the results described in your manuscript can be found. PLOS defines a study's minimal data set as the underlying data used to reach the conclusions drawn in the manuscript and any additional data required to replicate the reported study findings in their entirety. All PLOS journals require that the minimal data set be made fully available. For more information about our data policy, please see http://journals.plos.org/plosone/s/data-availability.

Upon re-submitting your revised manuscript, please upload your study’s minimal underlying data set as either Supporting Information files or to a stable, public repository and include the relevant URLs, DOIs, or accession numbers within your revised cover letter. For a list of acceptable repositories, please see http://journals.plos.org/plosone/s/data-availability#loc-recommended-repositories. Any potentially identifying patient information must be fully anonymized.

Important: If there are ethical or legal restrictions to sharing your data publicly, please explain these restrictions in detail. Please see our guidelines for more information on what we consider unacceptable restrictions to publicly sharing data: http://journals.plos.org/plosone/s/data-availability#loc-unacceptable-data-access-restrictions. Note that it is not acceptable for the authors to be the sole named individuals responsible for ensuring data access.

We will update your Data Availability statement to reflect the information you provide in your cover letter

**Answer:**

"All data is mentioned in the article. The tables and figures in this article contain all of the information provided. Construction industry workers were used in this study, and their information was completely confidential. Participants were not asked to provide their first or last names to ensure anonymity and reduce any potential bias in their responses. These measures were added to the methodology section and highlighted to provide clarity to readers. The inclusion of these measures was intended to address potential concerns related to participant privacy."

These items were added and highlighted in the method. These items were added to cover the latter.

**Comment:**

5. We note that you have stated that you will provide repository information for your data at acceptance. Should your manuscript be accepted for publication, we will hold it until you provide the relevant accession numbers or DOIs necessary to access your data. If you wish to make changes to your Data Availability statement, please describe these changes in your cover letter and we will update your Data Availability statement to reflect the information you provide.

**Answer:**

**There is no change in the data availability statement. All the data are mentioned in the article. The tables and figures in the article are the entire information of the article.**

**Comment:**

6. PLOS requires an ORCID iD for the corresponding author in Editorial Manager on papers submitted after December 6th, 2016. Please ensure that you have an ORCID iD and that it is validated in Editorial Manager. To do this, go to ‘Update my Information’ (in the upper left-hand corner of the main menu), and click on the Fetch/Validate link next to the ORCID field. This will take you to the ORCID site and allow you to create a new iD or authenticate a pre-existing iD in Editorial Manager. Please see the following video for instructions on linking an ORCID iD to your Editorial Manager account: <https://www.youtube.com/watch?v=_xcclfuvtxQ>

**Answer:**

**ORCID iD is announced.**

**Comment:**

7. Please include your full ethics statement in the ‘Methods’ section of your manuscript file. In your statement, please include the full name of the IRB or ethics committee who approved or waived your study, as well as whether or not you obtained informed written or verbal consent. If consent was waived for your study, please include this information in your statement as well.

**Answer:**

"All the workers who participated in this study completed a written consent form. The work steps were explained to the participants. The study protocol was reviewed and approved by the Research Ethics Committees of the School of Public Health & Neuroscience Research Center at Shahid Beheshti University of Medical Sciences in Tehran, Iran, with Approval ID: R. SBMU.PHNS.REC.1401.083. These items were added and highlighted in the methods section. We thank you for your attention and for reviewing our paper, and for providing valuable comments."

**Reviewer #1:** The manusript is technically correct. The applied statistics have been well described. the discussion is based on the results mentioned. Recommendations by the authors are evidence based. In my opinion, I believe that the mauscript may be accepted for publication

**Answer:**

Thank you for your opinion and kind attention, dear Reviewer. Dear Reviewer, I appreciate your insight and thoughtful consideration.

**Reviewer #2**: I would like to say that this piece of research aims to find factors affecting the non-use of safety harness at height among construction workers, has a comprehensive analysis to identify the potential and most influential factors out of all. But while reading the manuscript I found that it could be written in a much better way. I am not having the line numbers, so cannot point out the specific word or lines. Following are the comments to best of my knowledge:

**Comment:**

. 1. The language used in the manuscript is not technical, only use the language used in research papers. The English grammer should be checked with a skilful person as there are quite a few mistakes in the whole document. Check each and every statement thoroughly. Try to be consistant with choice of words , like, use 'factors affecting non-use...' and not 'reasons for not using...' as you have used word 'factors' in the title and aim.

**Answer:**

We thank you for your attention and reviewing our paper and presenting valuable and insightful comments**.** According to the opinion of the respected referee, corrections were made and highlighted in the text.

**Comment:**

2. .The introduction needs to be improved. Try not quote studies with authors names. Make your statements clear. Give rationale of your study in detail.

**Answer:**

Introduction improved. More literature review was added. These items were added and highlighted in the method .OSHA states that the construction industry is at the forefront in terms of defects in the standards. Accidents caused by falls from heights impose significant costs on both individuals and society. A full-body harness is a body support device that distributes forces between the wearer's shoulders, hips, and thighs. The harnesses have a D-ring in their design to help workers prevent falls and suspension. Moreover, a study by Angles demonstrated that the main reason for not using harnesses was the pressure on the workers' thighs and shoulders. Another study showed that most scaffolders were less willing to use the harness. Factors such as work pressure from managers, risk underestimation, and lack of training were identified as the main reasons. Bunney et al. also demonstrated that an unergonomic design reduces the motivation of workers to use harnesses at height. Wong et al. conducted a study on critical factors for the use or non-use of personal protective equipment among construction workers. They are influenced by personal, technological, and environmental factors. In their qualitative study, they conducted individual face-to-face interviews with Hong Kong construction workers to collect data. Goh and Sa'adon stated in their study that construction workers do not use harnesses due to the lack of supervisors' attitude and supervision. Hsiao stated that the reason for not using a harness is the lack of physical fit with the harness and the user's lack of comfort from the safety harness. The highlighted items have been added to the Methods section.

**Comment:**

3. .There seems no sequence in methods section. Give study design, area, population, tools, data collection, etc. in the appropriate manner.

**Answer:**

Construction workers were selected using convenience sampling, focusing on available samples in the north, west, east, and south of Tehran. These items were added and highlighted in the method. This is a qualitative study. Therefore, the sample size has been used to check the available samples and data saturation, as presented in the method. The interview was continued until the information obtained reached saturation, or in other words until no new information about the reasons for not using the harness was obtained after the interview.

.

**Comment:**

4. .Explain the data collection process in detail. Explain the selection proccedure of participants

**Answer:**

Construction workers were selected using convenience sampling, focusing on available samples in the north, west, east, and south of Tehran.

In this study, a semi-structured interview was used to identify the factors affecting the non-using of harnesses. The average, minimum, and maximum interview times were 30, 20, and 45 minutes, respectively. This time was chosen to manage the unreasonable answers of workers due to fatigue and boredom during the interview. One of the criteria for entering workers into the study was having at least one year of experience working at height in construction projects. Then, the workers were asked to explain the factors affecting the non-use of harnesses. With the consent of the workers, the interviews were recorded so that it was possible to review and reanalyze them. In the interview with the workers, they were asked to express their problems while using the harnesses and explain the reasons for not wanting to use it and express their feelings about the harnesses, what factors and suggestions they have for the comfort of harnesses, and what suggestions and solutions they have to improve the design of harnesses. New questions were raised based on the answers of some interviewees. Because this study was qualitative, the discussion continued until the required information was obtained. In this study, 23 workers were interviewed over ten days. After completing the interviews, the data were recorded, and MAXQDA software was used to categorize and code the data. These items were added and highlighted in the method.

**Comment:**

. 5. In SNA (2.3.1), it is written that the research focussed on the factors influencing the impact of non-using of harnesses. It is that way? I think the whole manuscript is about the factors affecting the non-use of harness and not influencing the impact of. Please make it clear in the aim of the study and be consitant till the conclusion.

**Answer:**

The main aim of the study was to identify the factors affecting the non-use of harnesses among construction workers, as mentioned in the Abstract and Methods sections. According to your comment, we have removed the impact of the manuscript as follows:

Therefore, this study focused on understanding the main factors influencing the non-use of harnesses among construction workers through a two-mode network as a quantitative technique. These items were added and highlighted in the method.

**Comment**

. 6. Give details of the tool used for semi-structured interview as it is not given anywhere.

**Answer:**

Based on the studies, some questions were prepared. Questions were asked to the workers and other questions arose from the answers of the workers that were asked to the people. The

Based on these studies, several questions have been prepared. Questions were asked to the workers and other questions arose from the answers of the workers that were asked to the people. The participants were asked the following questions:

What problems do you face when working with a harness?

Does the size of the harness fit your body size?

Do you think the harnesses are well-designed? What is your opinion about a better design of harnesses?

Do you feel comfortable with the harness you use?

How do you think a harness should be designed to make you feel more comfortable?

Do you always use harnesses? If the answer is no, explain the reason for not using the harness

In what cases do you prefer to not use harnesses?

What suggestions and solutions do you have to improve the design of this harness ?

These questions items were added and highlighted in the method

**Comment**

7. .Take second line of discussion to methods. Rewrite last four lines of first paragraph of the discussion section.

**Answer:**

The second line of discussion concerns the methods.In the present study, the factors affecting non-use the harnesses were investigated through a semi-structured interview (the average interview duration was 30 minutes). Four main factors influence the non-use of safety harnesses: design, comfort, management, and attitude. In the meantime, in terms of SNA analysis, the two influencing factors in non using restraints are related to non-ergonomic design and negative attitude of people.These items were added highlighted in the discussion

It was stated in the method.

**Comment:**

8. Make figure 2 clearer (quality wise) and simplify it, as it

**Answer:**

A: We tried to enhance the resolution of Figure 2 as much possible as. These items were added and highlighted in the result.

**Reviewer #3**

**Comment**

1. I feel the Introduction could be tightened up considerably? It seems to jump around quite a bit, so I found myself having to go back over previous sections to connect them to what I was reading. It is also repetitive.

**Answer:**

Introduction improved. More literature review was added. These items were added and highlighted in the method .OSHA states that the construction industry is at the forefront in terms of defects in the standards. Accidents caused by falls from heights impose significant costs on both individuals and society. A full-body harness is a body support device that distributes forces between the wearer's shoulders, hips, and thighs. The harnesses have a D-ring in their design to help workers prevent falls and suspension. Moreover, a study by Angles demonstrated that the main reason for not using harnesses was the pressure on the workers' thighs and shoulders. Another study showed that most scaffolders were less willing to use the harness. Factors such as work pressure from managers, risk underestimation, and lack of training were identified as the main reasons. Bunney et al. also demonstrated that an unergonomic design reduces the motivation of workers to use harnesses at height. Wong et al. conducted a study on critical factors for the use or non-use of personal protective equipment among construction workers. They are influenced by personal, technological, and environmental factors. In their qualitative study, they conducted individual face-to-face interviews with Hong Kong construction workers to collect data. Goh and Sa'adon stated in their study that construction workers do not use harnesses due to the lack of supervisors' attitude and supervision. Hsiao stated that the reason for not using a harness is the lack of physical fit with the harness and the user's lack of comfort from the safety harness. The highlighted items have been added to the Methods section.

**Comment:**
2. More description and related study in the field should be added for Social Network Analysis. It should be stated that this method of analysis is appropriate for this type of study.

**Answer:**

A: According to the comment, we added the following paragraph in the main manuscript.

The idea of this research was initiated in the philosophy of the SNA that focuses on the relationships between each pair of actors in a network, which specifies how important an actor in a network is. Thus, this research focused on revealing the main causes of influencing the non-use of the harness through the SNA analysis as a quantitative approach.

In this step, to gain an understanding of why the workers were unwilling to use the harness, semi-structured individual interviews were conducted. The data of the SNA consisted of two distinct sets of entities, which the construction workers are as actors and influencing causes on non-use of the harness as events. According to the affiliation matrix (table 1), the workers-causes interaction network, and each row of the matrix shows a worker’s affiliation with the influencing causes of non-use of the harness. This research used binary data (absent, i.e. 0.0, and present, i.e. 1.0) that "1" means that one cause is considered for the non-use of the harness by workers. In addition, a 0 (zero) indicated that was no selection by workers as an affected cause.

| Table 1. The two-mode network matrix for the workers and influencing causes on non-use of harness | | | | | | |
| --- | --- | --- | --- | --- | --- | --- |
| Actors | Events | | | | | |
|  | Cause1 | Cause2 | Cause3 | Cause4 | …. | Causen |
| Workers1 | 1 | 0 | 1 | 1 | … | 1 |
| Workers2 | 0 | 1 | 0 | 1 | … | 1 |
| Workers3 | 1 | 1 | 0 | 1 | … | 0 |
| Workers4 | 0 | 1 | 0 | 1 | … | 0 |
| …. | … | …. | …. | …. | … | …. |
| Workersn | 1 | 0 | 0 | 1 | … | 0 |

Comment 3. There needs to be a much more detailed description of the sample. How was the sample selected? How large were the organizations? What percentage of the workforce is included? What is the representation of the job categories presented?

Answer:

This is a qualitative study. Therefore, the sample size has been used to check the available samples and data saturation, as presented in the method. The interview was continued until the information obtained reached saturation, or in other words until no new information about the reasons for not using the harness was obtained after the interview.

**Comment**
4. The lack of any discussions of the practical implications of the findings, especially in light of the potential applicability of the research, is a significant weakness.

**Answer:**

The Discussion section has been revised according to the opinion of the reviewer.

These items have been added and highlighted in the discussion.

In the present study, the factors affecting non-use of the harnesses were investigated through a semi-structured interview (the average interview duration was 30 minutes). Four main factors influence the non-use of safety harnesses, which include design, comfort, management, and attitudinal factors. In the meantime, in terms of SNA analysis, the two influencing factors in non-use of restraints harnesses are related to non-ergonomic design and negative attitude of people. According to the findings of the present study, one of the reasons related to the design of the harness is the lack of use of soft materials in the harness, the complexity of wearing it, the lack of ergonomics, and the inappropriateness of the harness material. Non-ergonomic design was identified as one of the effective factors in the survey of workers. This can be attributed to employers' negligence in providing harnesses with user-friendly design. In the present study, attitudinal issues were raised as one of the main factors and groups in people's non-use of the harness, and factors such as the cumbersomeness of the harness, the limitation of work by wearing the harness, and its ineffectiveness were pointed out. In the present study, open questions were used in order to feel comfortable, express problems and develop the harness

**Comment**
5. References (in the text and reference list) should be prepared based on the journal format.

**Answer:**

References was prepared based on the journal format.

**Comment**
6. This paper would benefit from review by a native English speaker to assist with the sentence construction and spelling.

**Answer:**

This study uses a review by a native English speaker to assist with sentence construction and spelling.
7. The manuscript is scientifically incomplete and lacks a significant, novel contribution to the field.

Based on searches on scientific sites, very few studies have been conducted regarding the non-use of harnesses. The purpose and innovation of this study is to reveal the main causes influencing the non-use of restraint through the analysis of SNA as a focused quantitative approach. These items were added and highlighted in the Introduction.

Reviewer #4:

This is an interesting study. However, some major modifications are needed:
**Comment**:

1. there are several grammatical errors throughout the passage, for example somewhere "lake" is written instead of "lack",

**Answer:**

We thank you for your attention and reviewing our paper and presenting valuable comments.

Items were corrected.

**Comment:**
2. The introduction section is too short, a more extensive literature review is needed.

**Answer:**

Introduction improved. More literature review was added. These items were added and highlighted in the method. OSHA states that the construction industry is at the forefront in terms of defects in standards. Accidents caused by fall from height impose great costs on individuals and society. A full-body harness is a body support device that distributes forces between the wearer's shoulders, hips and thighs. The harnesses have a D-ring in their design to help the worker prevent fall and suspension .Moreover, the study by Angles demonstrated that the main reason for non using harnesses was the pressure on the workers' thighs and shoulders. Another study showed that most scaffolders were less willing to use the harness. Factors such as work pressure from managers, underestimation of risk, and lack of training were identified as the main reasons. Bunney et al. also demonstrated that an unergonomic design reduces the motivation of workers to use the harness at the height.Wong et al. in a study on critical factors for the use or non-use of personal protective equipment among construction worker .It is influenced by personal, technological and environmental factors. In their qualitative study, they used individual face-to-face interviews with Hong Kong construction workers to collect data. In their study, Goh and Sa'adon stated that construction workers do not use harnesses due to the lack of supervisors' attitude and supervision. Hsiao states the reason for non using the harness is the lack of physical fit with the harness and the user's lack of comfort from the safety harness.

**Comment:**
3. Authors wrote that a semi-quantitative approach is used. In this method there should be guide questions, which question did you use?

**Answer:**

In the interview with the workers, they were asked to express their problems while using the harnesses and explain the reasons for not wanting to use it and express their feelings about the harnesses.What factors and suggestions do they have for the comfort of harnesses and what suggestions and solutions do they have to improve the design of harnesses. The following questions were asked to the people.

(1) What problems do you face when working with the harness?
(2) Does the size of the harness fit your body size?
(3) Do you think the harnesses are well designed? What is your opinion about the better design of harnesses?
(4) Do you feel comfortable in the harness you are using?
(5) How do you think the harness should be designed to make you feel more comfortable?
(6) Do you always use a harness? If the answer is no, explain the reason for not using the harness
(7) In what cases do you prefer not to use a harness?

What suggestions and solutions do you have for improving the design of this harness.

These questions items were added and highlighted in the method

**Comment:**

4. How the participants were selected? They were workers, supervisors, or others? are all of them working at height? how experienced were they? How many participants did you interview?. all these issues should be addressed in the manuscript.

**Answer:**

Participants aged 20–50 years were included in this study. All the workers who participated in this study completed written consent forms. The work steps were explained to the workers. Construction workers were selected using convenient sampling focusing on available samples in the north, west, east and south of Tehran.

One of the criteria for entering workers into the study was having at least one year of experience working at height in construction projects. In this study, simple construction workers with at least one year of experience working with harnesses were selected. Construction supervisors do not work at height, only simple workers work at height. These items were added and highlighted in the method.

**Comment:**
5. please explain the stop point of interviews

**Answer:**

The interview was continued until the information obtained reached saturation, or in other words until no new information about the reasons for not using the harness was obtained after the interview. These items were added and highlighted in the work method.

**Comment:**

6. section 3.1 should be upgraded by examples from your own study. examples of complex coding and categorizing make this section more elaborate.

**Answer:**

Examples from previous studies and coding are also provided. These items were added and highlighted in the method.

In addition, in the study by Mardadi et al., the MAXQDA software was used to identify the causes of occupational neck pain in teachers [20]. Moreover, Turedi and Caylan categorized safety, security, and environmental issues based on participants' experiences in national marine policies using the MAXQDA software [21]. Alkhaleefah et al. used this software to promote transportation safety[22]. In addition, Salim et al. utilized MAXQDA to manage fires in public healthcare buildings[23].

According to the opinion of the respected Reviewer, the discussion section was revised.

These items were added and highlighted in the discussion.

**Comment:**

7. there are many types of harness and PFAS in the market, did you consider the type of harness used by the participants?

**Answer:**

In many construction workshops in Iran, harness is not used. In some construction workshops, the simplest type of restraint is also used. The results of our surveys showed that most people use the simplest type of harness, which is the full body harness class A for fall arrest, designed to protect the body during and after the fall arrest, they must have at least one fall arrest connection element that They should be explained in such a way that it is placed on the back in the middle of the rib cage, in the middle of the chest, approximately at the height of the sternum of the person who wears it. Simple harnesses are harnesses that a person does not work with in a suspended manner and only protects him in case of a fall. Therefore, under normal conditions, there is no pressure on the worker. We used this type of harnesses in this research and they do not have cushions and thick straps.

**Reviewer #5:**

**Comment:**

1. Why were 23 people selected?

**Answer:**

Since this part of the study was qualitative, the interview was continued until the information obtained reached saturation, or in other words, until a new code was obtained. In this study, 23 people were interviewed and no new information was obtained after 13 people. However, to ensure the results of the interviews, ten other people were also interviewed. In addition, to ensure the accuracy of the results, interviews were conducted with university professors. These items were added and highlighted in the method.

**Comment:**

2. What was question in the semi-structured interview?

**Answer:**

In the interviews with the workers, they were asked to express their problems while using the harnesses, explain the reasons for not wanting to use them, and express their feelings about the harnesses. What factors and suggestions do they have regarding the comfort of harnesses? What suggestions and solutions do they need to improve the design of harnesses?

**Comment:**
What characteristics did the selected people have?

**Answer:**

People in the age range of 20 to 50 years were included in this study. One of the criteria for entering workers into the study was having at least one year of experience working at height in construction projects.

**Comment:**

What were the inclusion and exclusion criteria?

**Answer:**

.

One of the criteria for entering workers into the study was having at least one year of experience working at height in construction projects. The exclusion criterion was a history of surgery in the abdominal, hip, and shoulder areas. These items were added and highlighted in the method.

**Comment:**
The demographic characteristics of the workers should also be mentioned, especially their level of education?
**Answer:**

We thank you for your attention and reviewing our paper and presenting valuable comments. Demographic information was added to the text. The average age of people was 30.6 ±5.2 and work experience in the construction industry was 8.37 ± 4.47. The average body mass index was 24.74±2.42. All the workers had an education level below diploma. 15% were illiterate, 45% could read and write, 20% had a bachelor's degree, 10% had a diploma, and 10% had a diploma. . These items were added and highlighted in the

**Comment:**
It is not clearly explained in method section. What was the input to the SNA model? Your study is qualitative. Where are the quantitative values entered to obtain the weights of the items in the model? Was it based on expert opinion?
**Answer:**
. In this step, to gain an understanding of why the workers were unwilling to use the harness, semi-structured individual interviews were conducted. The data of the SNA consisted of two distinct sets of entities, which the construction workers are as actors and influencing causes on non-use of the harness as events[29]. According to the affiliation matrix (table 1), the workers-causes interaction network, and each row of the matrix shows a worker’s affiliation with the influencing causes of non-use of the harness. This research used binary data (absent, i.e. 0.0, and present, i.e. 1.0) that "1" means that one cause is considered for the non-use of the harness by workers. In addition, a 0 (zero) indicated that was no selection by workers as an affected cause. These items were added and highlighted in the method.

**Reviewer #6:**

**Comment:**

The present study offers useful insights to identify the factors affecting the non-using of harnesses among construction workers in Tehran, Iran

**Answer:**

We thank you for your attention.

**Comment:**

1. Methods section- The authors have not mentioned the sampling strategy adopted to select the 23 workers. The sampling design has not been explicitly mentioned.

**Answer:**

.Since this part of the study was qualitative, the interview was continued until the information obtained reached saturation, or in other words, until a new code was obtained. In this study, 23 people were interviewed, and no new information was obtained after 13 people. But in order to ensure the results of the interview, 10 other people were also interviewed. Also, in order to ensure the accuracy of the results, interviews with university professors were also conducted in this regard. Construction workers were selected using convenient sampling focusing on available samples in the north, west, east and south of Tehran.

**Comment:**

3. Discussion section- Can the results of the study be generalized to other locations of Iran?

**Answer:**

The information obtained from this study can be generalized to Iran. Tehran is the capital and the most populous city in Iran. There are many construction projects in the city. In addition, the safety features in this city were greater than those in other cities. Therefore, the results of this research reflect the conditions in Iran. Due to a large number of construction activities in Tehran, people from other cities also refer to Tehran for employment; as a result, the results can be generalized to the whole of Iran. These items were added and highlighted in the Conclusion.

Thank you for your valuable comments.

Sincerely Yours

Dr. Mousa Jabbari
