# Supplementary material for: Identification and classification of factors affecting the non-use of safety harness at height among construction workers in Tehran
Source: PLoS One. 2023 Nov 30;18(11):e0287906. doi: 10.1371/journal.pone.0287906 (PMC10688616; doi:10.1371/journal.pone.0287906)
Supplement: Attachment — Submitted filename: Response to Reviewers (2).doc [file pone.0287906.s003.doc]

**Dear Mohammad Hossein Ebrahimi**

**Editorial Section Manager**

**PLOS ONE**

We thank you for your attention and reviewing our paper and presenting valuable comments.

We read the comments precisely and revised the manuscript. Some of the changes and answers to reviewers and editorial questions and comments are as follows:

**Reviewer #2**

**Comment:**

1. Although many comments have been addressed but it has been repeatedly asked to carefully check the whole manuscript for grammer and use only appropriate research language. The document still lacks that scientific writing. The staements are not connecting at many places and there are repetitions; whether it is introduction or conclusion.

There are many minor mistakes which should not be present at this point of time, like, the first paragarph of results has a repetition, please correct them.

**Answer:**

Thanks to the reviewer, the whole manuscript was re-checked for grammar.

Duplicate sentences like the following sentence were removed.

In this study, four main groups and twenty-seven sub-codes are determined regarding the unwillingness of workers to use of harnesses.

The average body mass index was 24.74±2.42. All the workers had an education level below diploma. 15% were illiterate, 45% could read and write, 20% had a bachelor's degree, and 10% had a diploma.

According to the opinion of the respected Reviewer, changes were made in the introduction and discussion for the purpose of greater coherence.

-----------------------------------------------------------

**Reviewer #3:**

**Comment:**

- Cohesion and coherence of sentences (paragraphs) should be considered in the introduction section.

- Relevant references should be added to sentences. For instance, in the introduction section, according to the previous explanations, according to the studies, and ... need references.

**Answer:**

According to the opinion of the respected Reviewer, references were added to sentences without references.

In this regard, qualitative studies could be used in interviews and software. Therefore, it is necessary to identify the critical factors and take corrective and preventive measures[24, 25].

These items were added and highlighted .

------------------------------------------------------------

**Reviewer #4:**

**Comment:**

The manuscript is acceptable based on the performed modifications. Howover, I can not approve that the manuscript is written and organized based on the journal guidlines.

**Answer:**

The manuscript was completed according to the format of the magazine and tried to apply all the desired items**.**

-----------------------------------------------------------

**Reviewer #7**

**Comment:**

• Is it better to mention the statistics of occupational accidents in Iran, especially falling from a height, in the introduction? And that it should be explained how the situation of such incidents is in Iran.

**Answer:**

Thanks for the comments of the respected Reviewer**,** Jabari (2016) also showed that 57% of the causes of death and disability in Tehran construction projects were due to falls from height. According to the statistics announced by the Social Security Organization of Iran in 2019, the number of 44,491 work-related accidents was recorded, of which 730 resulted in death. These items were added and highlighted in the Introduction.

**Comment:**

• It should be explained whether other articles, regulations and references have been checked in extracting the factors, or whether it has been considered only by experts? This is very important.

**Answer:**

The obtained factors were based on the opinions of workers, experts and professors.

According to the answers of some interviewees, new questions were raised. Since this study was qualitative, the discussion continued until the required information was obtained. The interview was continued until reaching data saturation, which means the lack of obtaining new information about the reasons for the non-use of the harness after the interview and the lack of obtaining a new code. In this study, 23 people were interviewed, and no new information was obtained after 13 people. But in order to ensure the results of the interview, 10 other people were also interviewed. Also, in order to ensure the accuracy of the results, interviews with university professors were conducted in this regard. In this research, 23 workers were interviewed for ten days. **These items highlighted in the method.**

**Comment:**

• More articles need to be compared in the discussion

**Answer:**

Thanks for the comments of the respected Reviewer**.**

In the ARTEAU study in 2018, he examined the selection of full-body harnesses according to the fit and comfort of harnesses in women. Women's discomfort in working with harnesses is due to the lack of attention of harness designers to women's physical dimensions.

Hsiao et al. (2003) reported that traditional anthropometric data are not suitable in addressing the harness problem.

In addition, traditional linear anthropometric data do not correspond well to harness components and are therefore not suitable for harness design in practice.

Gibbons found that the carpenters who used harnesses complained about the lack of fitness and were dissatisfied with body pressure Paying attention to ergonomic issues in the design of the harness is one of the main factors in the level of comfort of people and increases the usability of the harness among users.

These items were added and highlighted in the discussion.

**Comment:**

If possible, the quality of the Fig 2 should be improved.

**Answer:**

We tried to enhance the resolution of Figure 2 as much possible as. These items were added

**Comment:**

**•** In which of the factors is the position of the cost of buying harness considered?

• In which factors is the place of education and instructions important?

**Answer:**

The opinion of the respected Reviewer regarding the cost of the harness and training to use the harness is very valuable.

This article is a part of the harness design project, and the purpose of this study was to examine the lack of use of harness by workers from the perspective of experts. As a rule, according to Iran's labor law, the employer is obliged to provide personal protective equipment and teach how to use it correctly. And in this study, no person mentioned the lack of training regarding the use of harness.

Certainly, according to other studies such as Muhammad Fikri Hasmori et al.'s study entitled Causes for Lack of Usage of Safety Harness among Construction Workers in Malaysia: An Investigation, it has addressed more reasons.

**Comment:**

Inclusion & exclusion criteria are not clearly stated.

**Answer:**

The criteria for entering workers into the study was having at least one year of experience working at height in construction projects. The BMI of the people should be in the normal range. The age of the participants should be between 20 and 50 years. The exclusion criterion was having a history of any surgery in the abdomen, hip, and shoulder area. If the participants do not want to, they can leave the study during the study. In this study, people should have at least one year of experience using a harness, that is to know at least how to properly tie a harness and also have experience while working and wearing a harness in a real work environment. Because this can help a lot in conveying feelings and experiences. People should have a suitable BMI because people's obesity may affect people's bad feeling by the harness. And the age range of people was chosen between 20 and 50 years old, which is the age range for work. These criteria have also been used in studies.

These items were added and highlighted in the method.

**Comment:**

• The benefits of using the MAXQDA software in this study will be explained more. Of course, this is one of the strengths of the study, but it is better to explain it more.

**Answer:**

MAXQDA is a content analysis software used to organize and manage qualitative data. It helps in different stages of work including data collection, advanced data organization, help in data analysis, and displaying information and results in different ways. In this software, textual data are coded and classified through systematic processes. The use of software for coding qualitative data provides the possibility to simultaneously code codes, sub-codes and parts. Interviews and documents should be available in one space and be easily moved.

These items were added and highlighted in the method.

**Comment:**

• Ethical considerations need to be mentioned in detail.

**Answer:**

The study protocol was reviewed and approved by the Research Ethics Committees of the School of Public Health & Neuroscience Research Center - Shahid Beheshti University of Medical Sciences, Tehran. Iran (Approval ID: R. SBMU.PHNS.REC.1401.083).

The study was conducted in a quiet place.

Each worker was interviewed individually.

The confidentiality of workers' information and their opinions was maintained.

These items were added and highlighted in the method.

**Comment:**

• The abstract needs to be organized and completed.

**Answer:**

The abstract was organized and completed.

**Introduction** : accident of falling from a height is high among construction workers. Construction workers do not use harnesses. Thus, the present study was conducted to identify the factors affecting the non-use of harnesses among construction workers in Tehran, Iran.

**Materials and methods** In this study was conducted by interviewing professors and construction workers in order to identify factors affecting the non-use of harness. Factors influencing the non-use of safety harnesses were identified from the workers' point of view. The obtained data were classified and coded using MAXQDA 10 software. After that, the most essential, effective and powerful factors were identified using the degree and intersectionality of social network analysis.

**Results:** According to the interview results, 27 factors were determined as factors affecting non-use of harnesses by construction workers and divided into four main groups. The four groups were harness design, management factors, harness comfort, and attitudinal factors. Based on the results of the degree centrality, the non-ergonomic design and attitude of the harness inefficiency were identified as the most influential and powerful factors. The betweenness indicator also showed that the non-ergonomic design could mediate other factors in the non-use of the harness.

**Conclusion:** The findings showed that by considering various factors such as considering more comfort in the design of the ergonomic harness, it produced a better product. Also, the use of safety harnesses by workers increases.

**These items were added and highlighted in the abstract.**

**Comment:**

• The text needs to be edited by a language expert for ease of reading.

**Answer:**

The manuscript was re-edited by a language expert.

Thank you for your valuable comments.

Sincerely Yours

Dr. Mousa Jabbari
